# Supplementary material for: Mining Biosynthetic Gene Clusters of Pseudomonas vancouverensis Utilizing Whole Genome Sequencing
Source: Microorganisms. 2024 Mar 9;12(3):548. doi: 10.3390/microorganisms12030548 (PMC10974133; doi:10.3390/microorganisms12030548)
Supplement: Supplementary file 1 [file microorganisms-12-00548-s001.zip › microorganisms-2887864-supplementary.pdf]

Table S1: Five global protein families used in phylogenetic analysis.

SN	Protein family	Type	BRIC ID										
1	PGF_10357928	Proteins	fig|1124983.3.peg.615	fig|1136138.3.peg.4231	fig|1179778.3.peg.2229	fig|1207075.3.peg.503	fig|1218169.3.peg.5062	fig|1229486.3.peg.3270	fig|1330531.3.peg.4361	fig|205922.5.peg.565	fig|220664.5.peg.617	fig|237609.3.peg.4685	fig|95300.39.peg.4983
	PGF_10357928	Codons	fig|1207075.3.peg.503	fig|1218169.3.peg.5062	fig|1136138.3.peg.4231	fig|1179778.3.peg.2229	fig|1229486.3.peg.3270	fig|1330531.3.peg.4361	fig|220664.5.peg.617	fig|237609.3.peg.4685	fig|1124983.3.peg.615	fig|205922.5.peg.565	fig|95300.39.peg.4983
2	PGF_00413208	Proteins	fig|1124983.3.peg.1129	fig|1136138.3.peg.2780	fig|1179778.3.peg.906	fig|1207075.3.peg.4515	fig|1218169.3.peg.5780	fig|1229486.3.peg.2395	fig|1330531.3.peg.420	fig|205922.5.peg.1039	fig|220664.5.peg.1125	fig|237609.3.peg.4117	fig|95300.39.peg.2006
	PGF_00413208	Codons	fig|205922.5.peg.1039	fig|1330531.3.peg.420	fig|1136138.3.peg.2780	fig|237609.3.peg.4117	fig|1218169.3.peg.5780	fig|220664.5.peg.1125	fig|1124983.3.peg.1129	fig|1207075.3.peg.4515	fig|1179778.3.peg.906	fig|1229486.3.peg.2395	fig|95300.39.peg.2006
3	PGF_03050893	Proteins	fig|1124983.3.peg.3348	fig|1136138.3.peg.1592	fig|1179778.3.peg.3707	fig|1207075.3.peg.2921	fig|1218169.3.peg.7259	fig|1229486.3.peg.5358	fig|1330531.3.peg.2019	fig|205922.5.peg.2230	fig|220664.5.peg.3353	fig|237609.3.peg.1536	fig|95300.39.peg.4672
	PGF_03050893	Codons	fig|1136138.3.peg.1592	fig|205922.5.peg.2230	fig|1207075.3.peg.2921	fig|1229486.3.peg.5358	fig|220664.5.peg.3353	fig|1330531.3.peg.2019	fig|237609.3.peg.1536	fig|1218169.3.peg.7259	fig|1124983.3.peg.3348	fig|1179778.3.peg.3707	fig|95300.39.peg.4672
4	PGF_03790040	Proteins	fig|1124983.3.peg.1102	fig|1136138.3.peg.2760	fig|1179778.3.peg.597	fig|1207075.3.peg.983	fig|1218169.3.peg.4071	fig|1229486.3.peg.283	fig|1330531.3.peg.4817	fig|205922.5.peg.1012	fig|220664.5.peg.1099	fig|237609.3.peg.1083	fig|95300.39.peg.253
	PGF_03790040	Codons	fig|205922.5.peg.1012	fig|1207075.3.peg.983	fig|1218169.3.peg.4071	fig|1330531.3.peg.4817	fig|1124983.3.peg.1102	fig|1179778.3.peg.597	fig|237609.3.peg.1083	fig|220664.5.peg.1099	fig|1136138.3.peg.2760	fig|1229486.3.peg.283	fig|95300.39.peg.253
5	PGF_00026362	Proteins	fig|1124983.	fig|1136138.3.peg.	fig|1179778.3.peg.1	fig|1207075.3.peg.5	fig|1218169.3.peg.2	fig|1229486.3.peg.3	fig|1330531.3.peg.2	fig|205922.5.peg.53	fig|220664.5.peg.59	fig|237609.3.peg.49	fig|95300.39.peg.14

			3.peg.5828	3408	75	218	780	788	399	42	59	15	71
	PGF_00026362	Codons	fig|1124983.3.peg.5828	fig|1136138.3.peg.3408	fig|237609.3.peg.4915	fig|1229486.3.peg.3788	fig|1207075.3.peg.5218	fig|1330531.3.peg.2399	fig|205922.5.peg.5342	fig|220664.5.peg.5959	fig|1218169.3.peg.2780	fig|1179778.3.peg.175	fig|95300.39.peg.1471

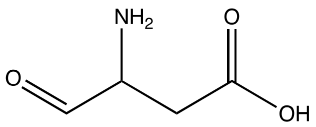

Cluster 2: 3-amino-4-oxobutanoic acid (NRPs)

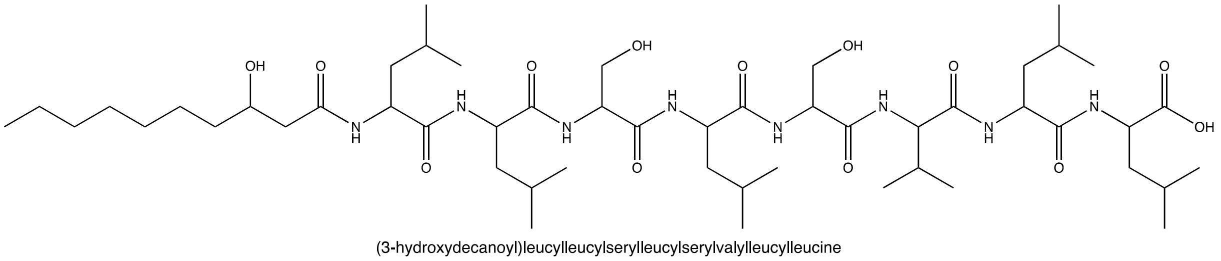

(3-hydroxydecanoyl)leucylleucylserylleucylserylvalylleucylleucine

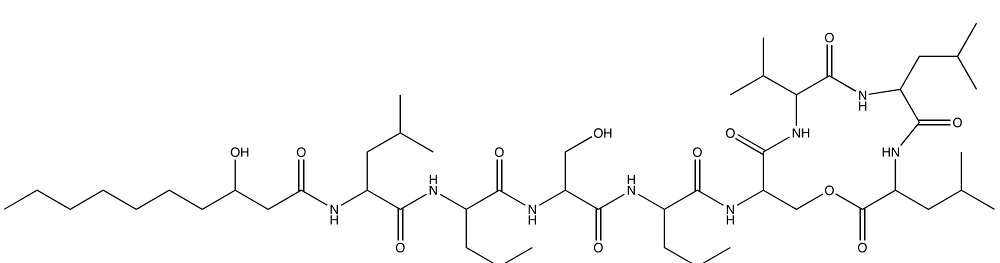

N-(1-((1-((1-((1-((3,6-diisobutyl-9-isopropyl-2,5,8,11-tetraoxo-1-oxa-4,7,10-triazacyclotridecan-12-yl)amino)-4-methyl-1-oxopentan-2-yl)amino)-3-hydroxy-1-oxopropan-2-yl)amino)-4-methyl-1-oxopentan-2-yl)amino)-4-methyl-1-oxopentan-2-yl)-3-hydroxydecanamide

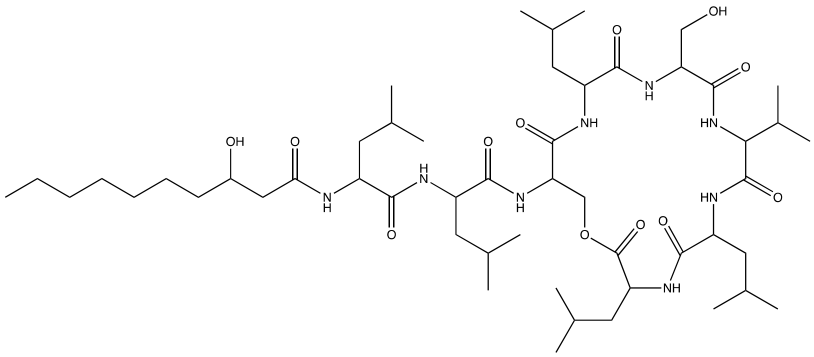

3-hydroxy-*N*-(1-((1-((12-(hydroxymethyl)-3,6,15-triisobutyl-9-isopropyl-2,5,8,11,14,17-hexaoxo-1-oxa-4,7,10,13,16-pentaazacyclononadecan-18-yl)amino)-4-methyl-1-oxopentan-2-yl)amino)-4-methyl-1-oxopentan-2-yl)decanamide

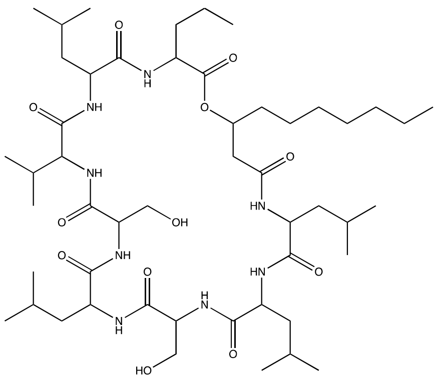

28-heptyl-12,18-bis(hydroxymethyl)-3,6,15,21,24-pentaisobutyl-9-isopropyl-1-oxa-4,7,10,13,16,19,22,25-octaazacyclooctacosane-2,5,8,11,14,17,20,23,26-nonaone

Cluster 3: NRPS

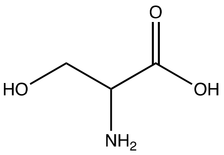

Cluster 4 and 12: Serine (NRPs)

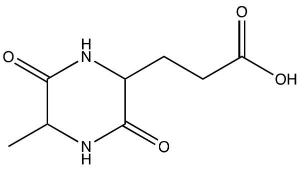

3-(5-methyl-3,6-dioxopiperazin-2-yl)propanoic acid
Cluster 7: Cyclodipeptide (XYP family)

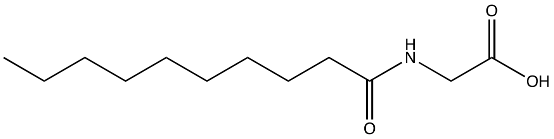

Cluster 8: Decanoylglycine (NRPS)

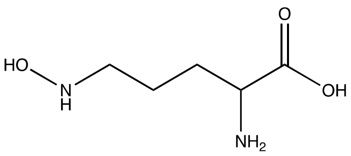

2-amino-5-(hydroxyamino)pentanoic acid
Cluster 9: NRPS

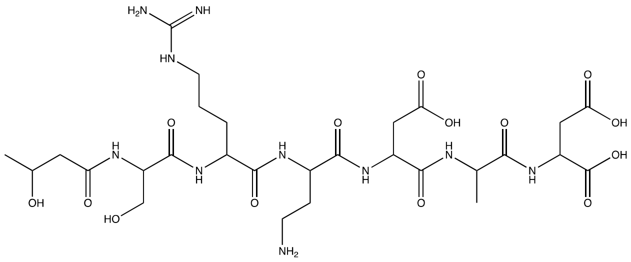

(4-amino-2-(5-guanidino-2-(3-hydroxy-2-(3-hydroxybutanamido)propanamido)pentanamido)butanoyl)aspartylalanylaspartic acid

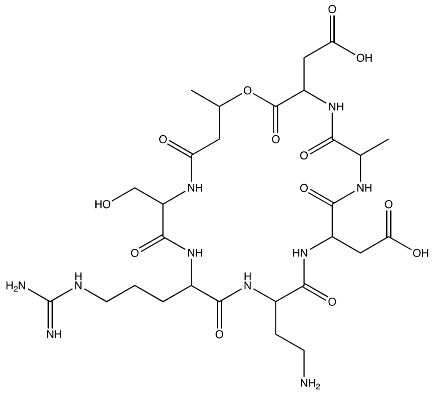

2,2'-(12-(2-aminoethyl)-15-(3-guanidinopropyl)-18-(hydroxymethyl)-6,22-dimethyl-2,5,8,11,14,17,20-heptaoxo-1-oxa-4,7,10,13,16,19-hexaazacyclodocosane-3,9-diyl)diacetic acid

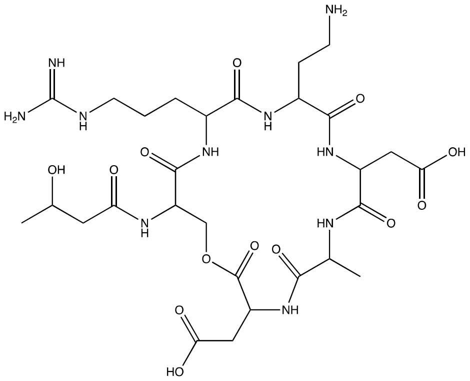

2,2'-(12-(2-aminoethyl)-15-(3-guanidinopropyl)-18-(3-hydroxybutanamido)-6-methyl-2,5,8,11,14,17-hexaoxo-1-oxa-4,7,10,13,16-pentaazacyclononadecane-3,9-diyl)diacetic acid

Cluster 10: NRPs

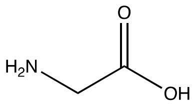

Cluster 11: Glycine (NRPS)

Figure S1: IUPAC name of the predicted secondary metabolites produced by *P. vancouverensis* using PRISM 4 prediction tool.
